# Increased SYK activity is associated with unfavorable outcome among patients with acute myeloid leukemia

**DOI:** 10.18632/oncotarget.4669

**Published:** 2015-08-11

**Authors:** Katalin Boros, Alexandre Puissant, Morgan Back, Gabriela Alexe, Christopher F. Bassil, Papiya Sinha, Eleni Tholouli, Kimberly Stegmaier, Richard J. Byers, Scott J. Rodig

**Affiliations:** ^1^ Department of Histopathology, Manchester Royal Infirmary, Manchester, UK; ^2^ Department of Pediatric Oncology, Dana-Farber Cancer Institute and Boston Children's Hospital, Boston, MA, USA; ^3^ INSERM U1065, Team 2, C3M, Nice, France; ^4^ The Medical School, The University of Manchester, Manchester, UK; ^5^ Department of Pathology, Brigham and Women's Hospital, Boston, MA, USA; ^6^ Department of Haematology, Manchester Royal Infirmary, Manchester, UK; ^7^ Institute of Cancer Sciences, The University of Manchester, Manchester, UK

**Keywords:** SYK tyrosine kinase, AML, poor prognosis, tissue microarray, clinical trials

## Abstract

Recent discoveries have led to the testing of novel targeted therapies for the treatment of acute myeloid leukemia (AML). To better inform the results of clinical trials, there is a need to identify and systematically assess biomarkers of response and pharmacodynamic markers of successful target engagement. Spleen tyrosine kinase (SYK) is a candidate therapeutic target in AML. Small-molecule inhibitors of SYK induce AML differentiation and impair leukemia progression in preclinical studies. However, tools to predict response to SYK inhibition and to routinely evaluate SYK activation in primary patient samples have been lacking. In this study we quantified phosphorylated SYK (P-SYK) in AML cell lines and establish that increasing levels of baseline P-SYK are correlated with an increasing sensitivity to small-molecule inhibitors targeting SYK. In addition, we found that pharmacological inhibition of SYK activity extinguishes P-SYK expression as detected by an immunohistochemical (IHC) test. Quantitative analysis of P-SYK expression by the IHC test in a series of 70 primary bone marrow biopsy specimens revealed a spectrum of P-SYK expression across AML cases and that high P-SYK expression is associated with unfavourable outcome independent of age, cytogenetics, and white blood cell count. This study thus establishes P-SYK as a critical biomarker in AML that identifies tumors sensitive to SYK inhibition, identifies an at-risk patient population, and allows for the monitoring of target inhibition during treatment.

## INTRODUCTION

Despite recent advances in the identification of mutational events responsible for the genesis and progression of AML, therapeutic options currently available for patients suffering from this disease remain inadequate. Although conventional chemotherapy achieves remission for a subset of patients, it is still the case that the majority of patients with AML relapse. Overall cure rates remain dismal, and the development of novel approaches to therapy is needed. A therapeutic alternative that has been developed, refined, and increasingly explored in recent years is kinase-targeted therapy. FMS-like tyrosine kinase 3 (FLT3), which undergoes an internal tandem duplication (ITD) in 35% of AML cases and is associated with poor prognosis, is one target thought to hold promise. The direct targeting of this kinase with a recently developed inhibitor quizartinib, for instance, has been shown to impair growth of FLT3-ITD-positive leukemia *in vitro* and in murine models and has induced dramatic responses in patients with AML [1]. Similarly, AZD1208, a potent and selective inhibitor of the PIM kinases, which are upregulated in AML, has demonstrated efficacy in preclinical models of AML [2]. We have identified spleen tyrosine kinase (SYK) as another candidate target in AML [3]. Targeting SYK activity in AML by pharmacological and genetic means induces differentiation and impairs growth *in vitro*, and it attenuates AML progression *in vivo*, an effect more dramatic in FLT3-ITD-positive AML [4–6].

One key to successful targeted therapy has been the ability to identify predictors of response (e.g., *HER2* amplification and herceptin, *BCR-ABL* rearrangement and imatinib, *ALK* rearrangements and crizotinib) while a second is the ability to measure target inhibition (e.g., loss of CRKL phosphorylation with imatinib treatment). In order to explore the role of activated SYK as a biomarker in patients with AML, determine whether SYK activity correlates with patient outcome, and evaluate the efficacy of targeting this kinase *in vivo* in AML blasts, a method is required for accurately measuring the levels of SYK activity in patient samples. Although flow cytometry is routinely used for analysis of cell surface markers in acute leukemia samples, intracellular flow cytometry is not presently validated for trial use in CLIA labs and cannot be undertaken retrospectively for survival analysis. Conversely, bone marrow trephine analysis, a key element in diagnosis and treatment assessment, lends itself to the study of archived samples with linked survival data, providing a distinct advantage over intracellular flow cytometric analysis for correlating biomarker activity with outcomes. We therefore chose to quantify P-SYK expression by immunohistochemistry in archival bone marrow trephine samples; this method can be easily adopted in any clinical pathology laboratory, a feature of importance for use in clinical management. In our hands, antibodies directed against SYK phosphorylated at position 525/526, which is both the kinase activity site of SYK and the target of pharmacological inhibitors, are not optimal for immunohistochemical staining [7–11]. We determined, however, that SYK phosphorylation at site Y323 parallels the phosphorylation level of the site Y525/526 in AML cell lines and that the IHC detection of the phosphorylated residue Y323 of SYK (P-SYK Y323) is a surrogate for SYK activity in AML cell lines. We applied this method to the study of 70 primary AML bone marrow biopsies and to the development of a critical assay for the reliable measurement of SYK activation in tumor tissue.

## RESULTS

### SYK activation is associated with response to small-molecule inhibitors of SYK

**Figure 1:**
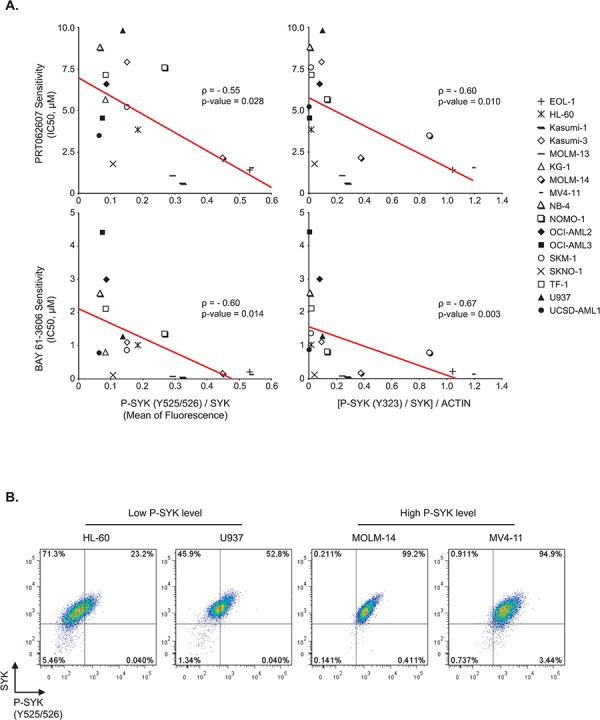
Association between level of SYK activation and response to small-molecule inhibitors of SYK **A.** Spearman correlation (ρ -score) between P-SYK (Y525/526)/SYK mean of fluorescence or [P-SYK (Y323)/SYK]/ACTIN and IC_50_s of 17 AML cell lines at day 6 post-treatment with two SYK inhibitors, PRT062607 and BAY 61-3606. **B.** Representative FACS plots for cell lines with high and low levels of P-SYK.

In order to establish whether the basal level of SYK activation is a good predictor of response to SYK targeting by small-molecule inhibitors in AML, we used flow cytometry and western blots to evaluate the basal level of SYK expression and phosphorylation of sites Y525/526 and Y323 in a panel of 17 AML cell lines. Then, we determined for each of these cell lines the IC50 corresponding to two different SYK inhibitors, PRT062607 and BAY 61-3606. As shown in Figure 1A, the more elevated the P-SYK/SYK ratio, the lower the half maximal inhibitory concentration required for each SYK inhibitor (ρ-score = −0.55 and −0.60 for P-SYK (Y525/526) with PRT02607 and BAY 61-3606 respectively, and ρ-score = −0.60 and −0.67 for P-SYK (Y323) with PRT02607 and BAY 61-3606). Cell lines with low P-SYK/SYK ratios were less sensitive to the effects of these inhibitors. Our results suggest that the basal level of SYK activation is a good index of response to SYK inhibitors. FACS plots are shown in Figure 1B for cell lines with high and low levels of P-SYK, which were then selected alongside several other cell lines with similar phosphorylation profiles by flow cytometry as a representative model for further study of SYK activation in AML.

### P-SYK Y323 is an IHC-detectable marker for SYK activity in AML

**Figure 2:**
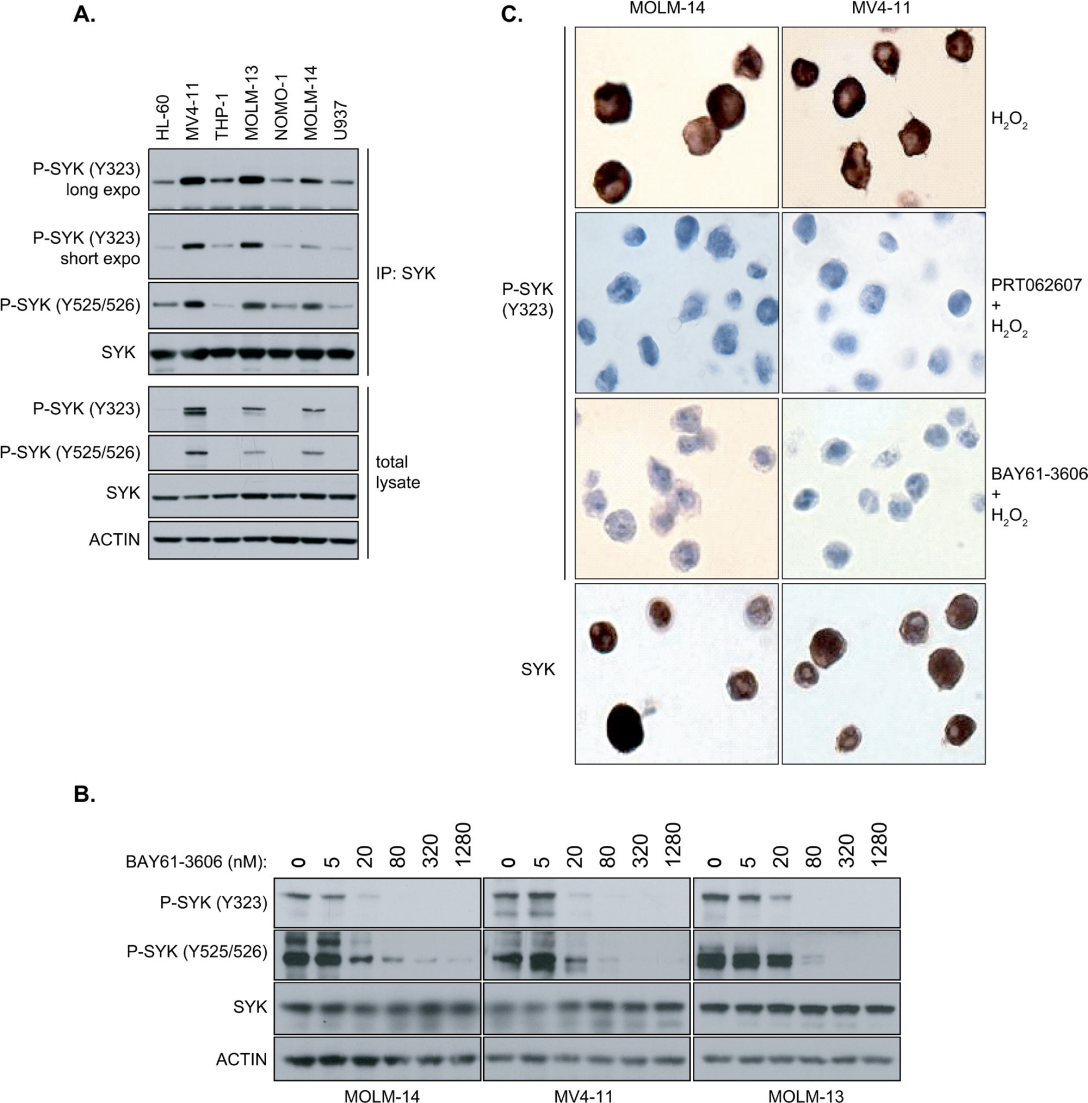
Levels of phosphorylation of Y323 and Y525/526 sites on SYK with and without BAY61-3606, a SYK inhibitor, in AML cell lines **A.** SYK kinase was immunoprecipitated (IP) from lysates of various AML cell lines using an anti-SYK-specific antibody. Immunoprecipitated SYK was analyzed by immunoblotting (IB) using antibodies specifically directed against phosphosites Y323 and Y525/526. Total lysate was immunoblotted using the same phospho-SYK (Y323 and Y525/526) antibodies. SYK and Actin were used as loading controls. **B.** MOLM-14, MV4-11 and MOLM-13 AML cell lines were treated for 6 hours with increasing concentrations of BAY61-3606, a selective SYK inhibitor. Total lysates were analysed by immunoblotting using antibodies specifically directed against phospho-sites Y323 and Y525/526 of SYK. Total SYK and Actin were used as loading controls. **C.** MOLM-14 (left column) and MV4-11 (right column) AML cell lines were treated with H_2_O_2_ to stimulate SYK phosphorylation and with (middle row) or without (top row) the SYK inhibitors, BAY61-3606 and PRT062607. After fixation, cells were then stained for P-SYK (Y323) or total SYK (bottom row).

To test whether SYK phosphorylation at the Y323 site is a reliable marker for SYK activity in AML, we compared the level of SYK phosphorylation at the Y323 site with the canonical Y525/526 site, which has been well defined as a predictor for SYK activity [7]. As shown in Figure 2A, the level of SYK phosphorylation at Y323 parallels that detected at Y525/526. To more definitively validate that detection of the phosphorylated Y323 site provides the same read-out for SYK activation as the Y525/526 site, we treated AML cell lines exhibiting the highest levels of phosphorylation at Y323 and Y525/526 with increasing concentrations of the SYK inhibitor BAY61-3606 (Figure 2B). In each cell line, BAY61-3606 markedly reduced the SYK phosphorylation level at both sites, suggesting that Y323 phosphorylation tracks with that of Y525/526 and thus can serve as a surrogate for SYK phosphorylation, activation, and response to small-molecule inhibition.

Because routine measurement of P-SYK activity at site Y525/526 is hampered by the lack of efficient IHC-grade Y525/526-directed antibodies, these results highlighted Y323 as an attractive candidate target for the detection of SYK activation by IHC. To test this hypothesis, MOLM-14 and MV4-11 cells were treated with BAY61-3606 or PRT062607 prior to fixation and processing with paraffin wax for IHC using anti-P-SYK Y323 antibody (Figure 2C). Because IHC is a less sensitive technique than western blotting, H_2_O_2_ was used to stimulate SYK phosphorylation. The positive staining for phosphorylated SYK at residue Y323 that was observed at baseline was abolished with treatment by either compound, thus validating the anti-phospho-Y323 antibody to assay SYK activation by IHC.

### SYK exhibited different levels of activation in bone marrow from primary patients with AML

**Figure 3:**
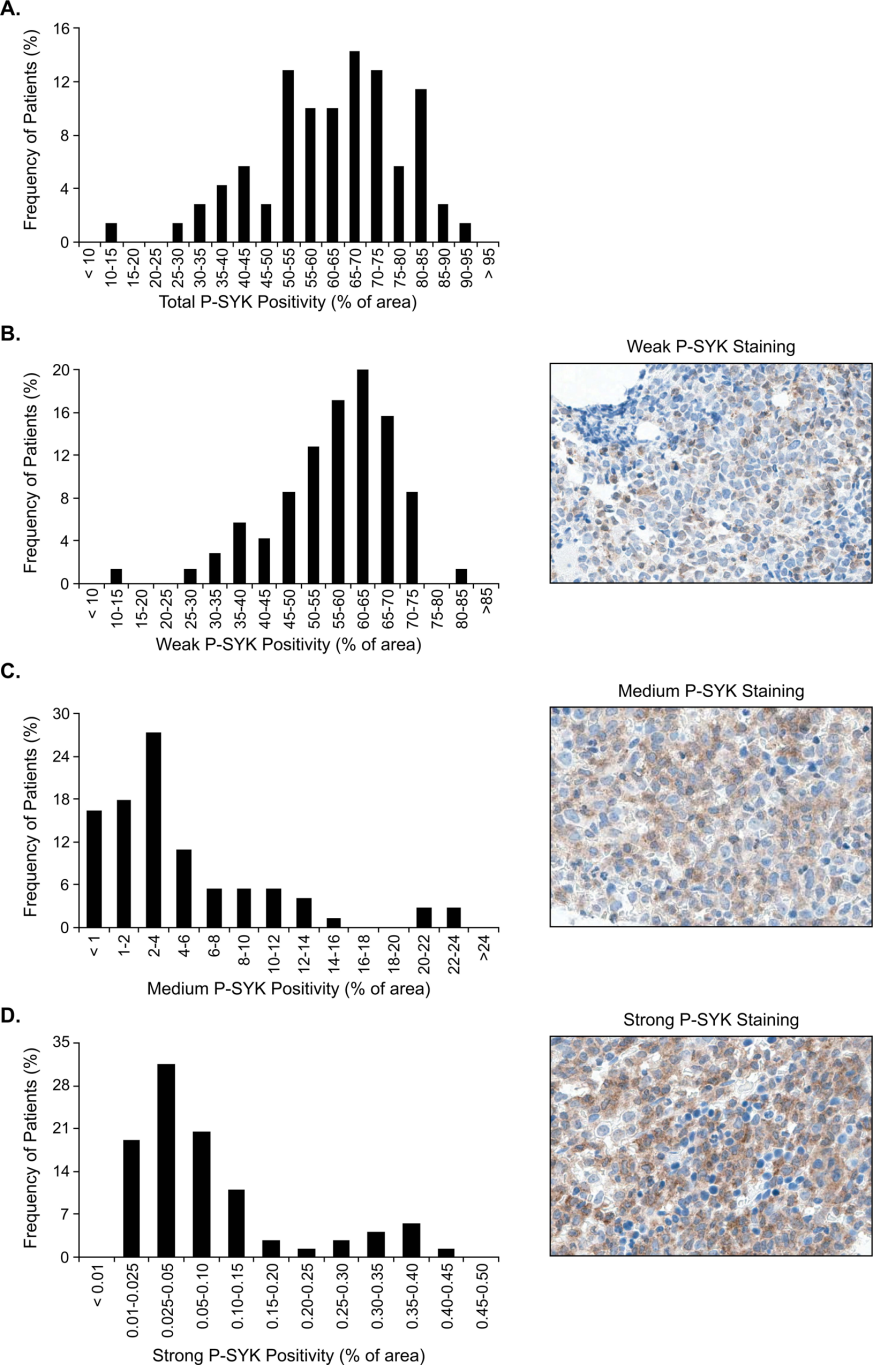
Frequency distribution of P-SYK positivity The x axis indicates intervals of P-SYK positivity for **A.** total (any intensity value > 0), **B.** weak/1+, **C.** medium/2+ and **D.** strong/3+ staining intensity. Insets show representative images of the respective staining intensities. Frequency of cases in each interval is shown along the y axis. P-SYK staining was detected in every sample, with at least low percentage of weak staining intensity. Measurements were performed using the Aperio Image Analysis system. Micrographs were imaged using a Nikon Eclipse 80i microscope, with a Nikon Plan Apo 40_/0.95 air objective and captured using a Nikon DS-F digital camera with NISElements D 3.1 software, with manipulation in CorelDRAWX3.

A cohort of 70 patient AML samples was screened by IHC using this validated anti-Y323 P-SYK antibody to determine the level and profile of SYK activation in bone marrow biopsies from patients with AML (Figure 3). Although total SYK expression level was uniformly and strongly positive in all samples (Supplemental Figure 2, and data not shown), the SYK phosphorylation level was variable both within and between samples, with at least some low percentage areas of weak staining in all cases (Figure 3A). Moreover, the pattern of staining across all patients was cytoplasmic and varied from isolated positive cells to focal sheets of cells.

**Figure 4:**
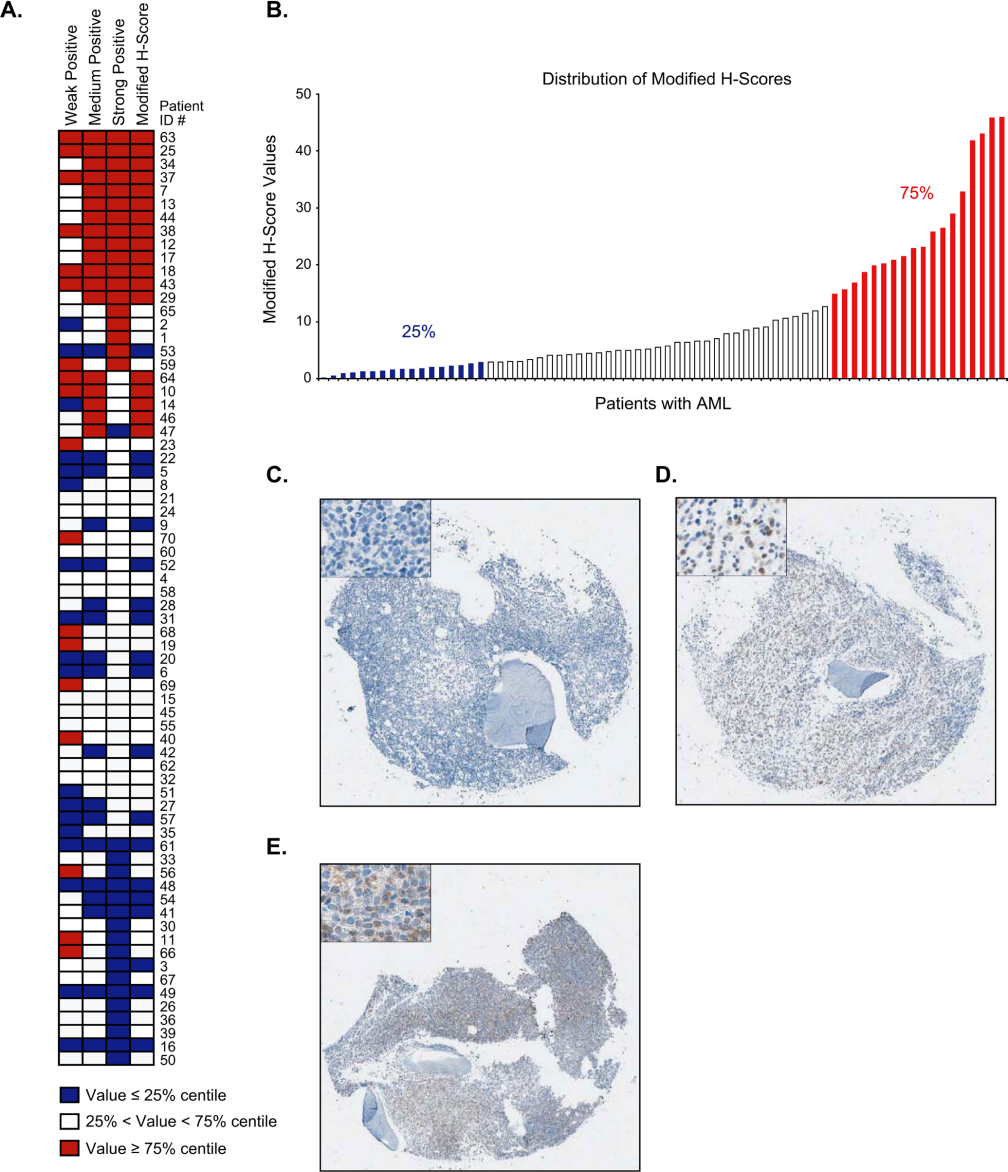
Distribution and association of P-SYK expression levels and modified H-scores **A.** Heatmaps showing the relationships between total, weak, medium, and strong P-SYK staining intensities along with H-score and modified H-score values grouped below the 25%, between 25 and 75% and above 75% groups in each individual patient sample. The medium, strong and modified H-score values track together. **B.** Bar graph showing modified H-scores of individual cases, in ascending sequence. Colors indicate the cut-off values for the 25% (blue) and 75% (red) groups. C-E: Representative cores showing P-SYK staining with modified H-scores below **C.** around **D.** and over **E.** the 75% cut-off values. Images were taken as screenshots of the Aperio Spectrum scanned TMA slides at 4x magnification; insets show representative areas of the screen shots at 20x magnification.

Given the broad range of P-SYK signal detected in the samples, P-SYK staining intensity was scored as weak (1+ positivity), medium (2+ positivity) and strong (3+ positivity) using standard imaging software. The percentage of representation of each of these categories was evaluated for each sample. As shown in Figure 3B–3D, the weak P-SYK staining represented approximately 14% to 80% (median 59.06%, SD 12.53) of the area in each sample, the medium P-SYK staining from 1% to 22% (median 2.78%, SD 5.56), and the strong P-SYK staining from 0.01% to 0.4% (median 0.049%, SD 0.11). As expected, a high correlation (r^2^ = 0.708, *p* < 0.0001) between the percentages of area was observed between medium and strong (2+ and 3+ positivity) staining, suggesting that these two categories are highly associated in each patient sample (Supplemental Figure 3). This analysis is depicted in the heatmap in Figure 4A where we observed a higher association per sample of medium and strong positive staining in comparison with weak staining, which exhibited a correlation score of only 0.22 and 0.101 with medium and strong staining respectively (Supplemental Table 3). Therefore, as a summation of P-SYK staining, we devised a modified H-score which included areas with medium (2+) and strong (3+) staining and omitted areas with either no (0) or weak (1+) staining. As demonstrated by the heatmap, the modified H-score values robustly mirrored the P-SYK intensity values observed either in medium, strong or both categories combined. When applied to all cases, the modified H-score revealed a broad distribution of total tumor staining across samples (Figure 4B); nevertheless, division at the 75^th^ percentile allowed for a clear distinction between cases with robust P-SYK activation among the tumor cells and those without (Figure 4C–4E).

### SYK activation in bone marrow from primary AML samples is associated with poor patient survival

**Figure 5:**
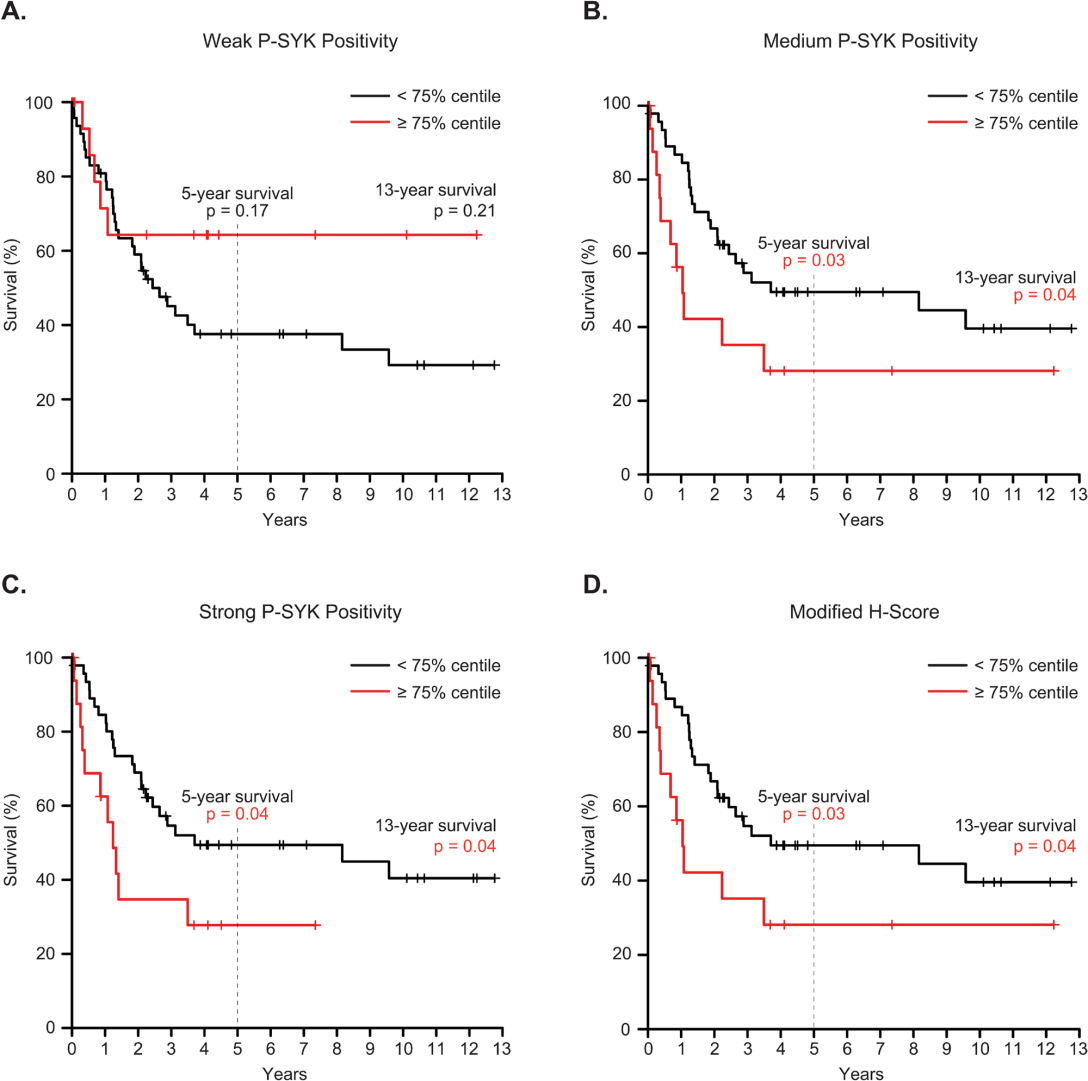
Association between P-SYK expression levels and clinical outcome Kaplan-Meier analysis was performed using different intensity P-SYK staining grouped either below or equal to or above the 75% staining frequency values for **A.** weak, **B.** medium, **C.** strong P-SYK staining, or **D.** the modified H-score. *P* values for the individual analyses are indicated for 5- and 13-year survival.

We next tested whether this method of sample categorization based on level of SYK activation could be used as a tool to predict patient outcome. We divided patient samples into two subgroups: one below and one greater than or equal to the 75% distribution of the samples for weak, medium, strong, or combined medium and strong P-SYK staining. A multivariate Cox regression survival analysis performed on these two subgroups revealed that medium P-SYK level was found to be an independent prognostic factor (*p* = 0.02), as was strong (*p* = 0.05), and combined medium and strong P-SYK level (*p* = 0.02) (Table 1); similarly the modified H-score split at the 75% centile was also an independent prognostic factor (*p* = 0.02). Thus, as visualized on the Kaplan-Meier curves in Figure 5A–5D, while weak staining for P-SYK above or below the 75% centile was not associated with outcome (*p* = 0.17), patients exhibiting medium or strong staining for P-SYK equal to or above the 75% were associated with unfavorable outcome compared to the other subgroup (*p* ≤ 0.04). Similarly, a modified H-score that groups medium and strong staining greater than the 75% cut-off was associated with a worse outcome (*p* = 0.04) (Figure 5D). Detailed information on the clinical characteristics of patients with medium, strong and both combined P-SYK staining above the 75% is shown in Supplemental Table 1. Finally, the same multivariate Cox regression analysis showed no significant link between either medium, strong or both combined P-SYK level and other covariates, such as age at diagnosis, cytogenetic subtype, FAB subgroup or white blood cell count (Table 1). This was confirmed by Kruskal-Wallis test, which demonstrated no significant correlation between medium and strong staining and each of these categories (not shown).

**Table 1:** Summary of results of multivariate Cox regression analyses

Covariate	*P* value	Covariate	*P* value	Covariate	*P* value
% Strong, 75^th^ centile	**0.05**	% Medium, 75^th^ centile	**0.02**	% Medium and Strong, 75^th^ centile	**0.02**
Age	0.66	Age	0.72	Age	0.72
Cytogenetics	0.96	Cytogenetics	0.73	Cytogenetics	0.73
FAB group	0.18	FAB groups	0.24	FAB groups	0.24
White Cell Count	0.10	White Cell Count	0.11	White Cell Count	0.11

Categorical P-SYK staining intensities and modified H-score were included together with age at diagnosis (Age), cytogenetics, FAB group and white cell count at diagnosis in multivariate Cox regression analyses.

## DISCUSSION

The identification of novel druggable targets in hematological malignancy requires the development of routinely applicable assays for the assessment of target levels and/or activation. These assays are essential for patient selection, subsequent treatment stratification, and the measurement of target inhibition by the therapeutic intervention in question. In particular, clinical trials should be conducted using the same assays for patient selection and response monitoring that will eventually be adopted for routine clinical use. A necessary first step in the evaluation of SYK inhibitors in clinical trials involving patients with AML would thus involve the measurement of P-SYK in routine clinical samples, preferably using a method amenable to widespread adoption.

Although flow cytometric immunophenotyping against cell surface antigens is a useful tool in diagnostic hematopathology, this approach remains insufficient for certain clinical applications. Moreover, while intracellular flow cytometry now allows for the measurement of changes in phosphorylation states, this approach can be difficult to apply to a multi-institutional clinical trial where samples are shipped, potentially altering the highly dynamic phosphorylation state [13]. This technical limitation is not observed in IHC and moreover, paraffin immunohistochemical immunophenotyping offers preservation of tissue architecture and allows for the adequate analysis of isolated or clustered cells, such as those considered positive for strong P-SYK staining in the present analysis. Thus, the IHC method for SYK activation in AML described in this paper can be standardized and performed on routine samples. It therefore holds promise as an assay to be deployed for research purposes in clinical trials, and subsequently, it can be rolled out as a routine diagnostic to guide molecularly informed therapy. An important question when evaluating the IHC staining of phosphorylated proteins involves the preservation of phosphorylation status during the fixation process. This is particularly relevant in the case of surgical resection specimens in which there is both warm and cold ischemic time and for which the penetration of the tissue by the fixative, usually formalin, is unequal, leading to the variable preservation of the phosphorylation status of proteins [14–17]. These concerns are mitigated by the protocol developed in this study, which utilizes bone marrow trephine samples placed immediately in formalin after collection with minimal ischemic time. Moreover, the requirement for only a small amount of sample enables rapid and uniform fixation. The fact that all samples were EDTA-decalcified in a single lab according to a standard protocol that was static over the course of the study reduced variation due to artefact and loss of phospho-sites to a minimum.

The high degree of fidelity demonstrated by our P-SYK-directed IHC assay is complemented by its ability to discriminate between varying degrees of P-SYK activation. The use of modified H-scores as described here highlights a clear difference between samples with very low P-SYK activation and those whose levels are more significantly elevated, indicating that P-SYK lends itself to assessment by visual methods such as IHC. Although both the selection of regions of tissue for evaluation and the method of data analysis present other considerations, large areas potentially prone to artefact can be excluded manually prior to the data analysis, and automated analysis and scoring of scanned tissue slides has been shown to produce results concordant to those produced by experienced pathologists [12, 18]. The sensitivity and specificity of detection assays can also be increased with computational methods such as the deconvolution analysis algorithm presented in this study. In this case, the clear difference between low and high frequencies of medium P-SYK expression, recapitulated in the “jump” of the modified H-scores shown in the distribution plot (Figure 4B), provides reliable evidence that the data are adequately robust to counteract analytical compromises related to assay sensitivity.

In addition to these general concerns, IHC-based assays directed against the activation levels of specific kinases may face issues specific to the kinase in question itself at some point during the development process. With regards to P-SYK, for example, in our hands, antibodies directed against the canonical activation site Y525/526 are suboptimal for immunohistochemical staining, which necessitated the identification of a proxy site for activation that could be detected more easily by IHC. The Y323 phosphorylation site has proved easier to assay than other previously described epitopes, and commercial availability of Y323 phospho-specific antibodies will facilitate the translation of these results to clinical practice. Furthermore, the parallel fashion in which the Y525/526 site and the Y323 site are phosphorylated and dephosphorylated in the absence and presence of a small-molecule inhibitor of SYK, BAY 61-3606, suggests that the Y323 site is indeed a valid proxy for Y525/526.

The development of a reliable, standardized protocol for evaluating the level of activation of target kinases in clinical samples is only as useful as the diagnostic marker in question. SYK, for example, has been implicated in the pathogenesis of B-cell and T-cell lymphomas and certain myeloid malignancies such as myelodysplastic syndrome (MDS) [19, 20] and AML [3, 6, 21]. The development of IHC assays focused on the detection of SYK activation in primary specimens will enable the establishment of SYK as a diagnostic marker in various diseases. Although strong staining was a relatively rare event (<1% of total tumor area), for instance, the presence of a higher percentage of tumor positivity for strong staining was associated with worse outcome when it did occur. A high percentage of medium intensity was similarly associated with poor outcome, which was maintained when the percentage of medium and strong positive staining were combined, indicating that even a modest increase in P-SYK activation is associated with poor outcome. That a margin as slight as this can have such dramatic consequences for patients is indeed surprising, but prior research indicates that cellular populations within patients are highly heterogeneous [22, 23]. It is thus possible that within the broad distribution of P-SYK observed by IHC in the bone marrow cores, the presence of medium and strong P-SYK staining reflect a subset of cells which have strong survival/proliferative signalling and thus behave with greater aggression. Recent investigations into the mechanism of SYK activation in AML have linked SYK to integrin beta signalling and the oncogenic transcription factors STAT3 and STAT5 [4, 5], providing a possible explanation for the clustered patterns that cells with high P-SYK activation exhibited under our IHC assay. Whether the presence of medium and strong P-SYK staining and/or its association with poor outcome is independent of other molecular features remains to be determined in a prospective study because this historical collection of AML samples lacked modern annotation for molecular features.

Taken as a whole, these results establish for the first time the frequency of P-SYK activation in a large cohort of FFPE AML bone marrow samples using an IHC-based assay. This approach is capable of determining the presence and degree of SYK activation and should serve in the future as a clinically useful method for the measurement of this target as part of clinical trials and for implementation into subsequent routine use.

## MATERIALS AND METHODS

### Cell Culture

HL-60 and U937 cell lines were purchased from the American Type Culture Collection. THP-1, MOLM-14 and MV4-11 were kindly provided by Dr. Scott Armstrong. NOMO1 and MOLM-13 were kindly provided by Dr. Ross Levine. All cell lines except MOLM-13 cells were maintained in RPMI 1640 (Cellgro, Manassas, VA, USA) supplemented with 1% penicillin-streptomycin and 10% fetal bovine serum (FBS, Sigma-Aldrich, St Louis, MO, USA) at 37°C with 5% CO_2_. MOLM-13 were maintained in IMDM (Gibco, Grand Island, NY, USA) supplemented with 1% penicillin-streptomycin and 10% FBS.

### Chemicals

PRT062607 (Selleckchem) and BAY 61-3606 EMD Biosciences (Billerica, MA, USA) were dissolved in dimethyl sulfoxide (DMSO) and stored at −20°C.

### Immunoprecipitation

Cells were suspended in lysis buffer (50 mM Tris-HCl [pH 7.4], 150 mM NaCl, 20 mM EDTA, 50 mM NaF, 0.5% NP-40, 1 mM dithiothreitol) supplemented with EDTA-free protease inhibitors and PhoSTOP phosphatase inhibitors (Roche, Mannheim, Germany). Lysates (500 μL) were then incubated with 2 μg antibody and 35 μL protein G–Sepharose (Invitrogen, Carlsbad, CA, USA) at 4°C overnight. Beads were washed 5 times with 1 mL lysis buffer before boiling in Laemmli sample buffer and performing SDS–polyacrylamide gel electrophoresis (PAGE), transfer to nitrocellulose membranes, and immunoblotting.

### Immunoblotting

Whole-cell lysates were extracted in 1 × Cell Lysis Buffer (Cell Signaling, Boston, MA, USA) supplemented with EDTA-free protease inhibitors and PhosSTOP phosphatase inhibitors (Roche). Western immunoblotting was performed as follows. Briefly, 10–50 μg of protein was loaded on an SDS-PAGE gel and transferred to a 0.45 μM nitrocellulose membrane. Membranes were blocked in 5% bovine serum albumin (BSA; Sigma-Aldrich) in Tris-buffered saline with 1% Tween-20 (TBST), incubated in primary antibody overnight at 4°C, incubated in secondary antibody for 2 hours at room temperature, and developed using Western Lightning ECL (Perkin Elmer, Waltham, MA, USA). Primary antibodies against total SYK and Actin were purchased from Santa Cruz Biotechnology. The phospho-SYK (Y323) primary antibody was obtained from Epitomics (now Abcam, Cambridge, MA, USA) and the phospho-SYK (Y525/526) from Cell Signaling (Boston, MA, USA).

### Flow cytometry

Cell lines were washed twice with PBS-0.1%BSA-2 mM EDTA before a 20-minute fixation with BD Cytofix/CytoPerm Fixation and Permeabilization Solution (BD Biosciences). Cells were then washed three times with BD Perm/Wash Buffer and incubated for 45 minutes at 4°C with a combination of anti-human PE-conjugated P-SYK (Y525/526) (used at 1/25, Cell Signaling) and APC-conjugated SYK (used at 1/100, eBioscience), or the corresponding combination of isotype control antibodies. Cells were washed three times in Perm/Wash Buffer and analyzed using a BD FACSCanto II analyzer.

### Viability assay

Viability experiments were performed in 384-well format in seven replicates per dose using the Promega Cell-Titer Glo ATP-based assay per the manufacturer's instructions. Leukemia cell line viability was evaluated at day 6 post-treatment with PRT062607 and BAY61-3606 in a two-fold dilution to establish the drug concentrations that reduced cell viability to 50 percent of the vehicle controls (IC50). Values for IC50 were calculated by interpolating a natural cubic spline fit to the measured viability data in R (using the spline function).

### Immunohistochemistry

Cell lines MV4-11 and MOLM-14 were treated with H_2_O_2_ to induce high levels of SYK activation and were then treated with the SYK inhibitor BAY 61-3606. Cells were harvested, pelleted by centrifugation, fixed in 10% neutral buffered formalin, and processed to paraffin wax, following which sections were cut and stained by IHC for Y323 P-SYK. Slides were baked, soaked in xylene, passed through graded alcohols, and then pretreated with DAKO pH9 retrieval solution (DAKO, Carpinteria, CA, USA) in a steam pressure cooker (Decloaking Chamber; BioCare Medical, Concord, CA, USA) as per the manufacturer's instructions, followed by washing in distilled water. All further steps were performed at room temperature in a hydrated chamber. Slides were treated with peroxidase block (DAKO) for 5 minutes to quench endogenous peroxidase activity. Staining was conducted with 1:100 anti-pY323 SYK (Epitomics, Inc., EP574-3; now Abcam, Ab62338, Cambridge, MA, USA) for 1 hour, followed by washing and staining with rabbit ENVISION (DAKO) and diaminobenzidine (DAB) chromogen kit (DAKO) as per the manufacturer's instructions. As shown in Supplemental Figure 1 for 6 representative patient samples, immunohistochemical staining for P-SYK was reproducible across duplicate staining runs. Slides were counterstained with Harris haematoxylin (Polyscientific, Bay Shore, NY, USA). Micrographs were imaged using a Nikon Eclipse 80i microscope, with a Nikon Plan Apo 40_/0.95 air objective and captured using a Nikon DS-F digital camera with NISElements D 3.1 software, with manipulation in CorelDRAWX3.

### Sample collection

Presentation bone marrow trephine samples from 70 patients diagnosed with AML between 1994 and 2005 were retrieved from the histopathology archives at Manchester Royal Infirmary. These patients were selected on the basis of tissue availability from a larger group of 192 patients previously selected from the archives as detailed in Byers et al. [12]. All trephine biopsy samples were handled identically and were routinely processed, formalin-fixed, paraffin-embedded and EDTA-decalcified at presentation. All material used was residual, anonymized diagnostic tissue, with consent for its use in research granted (REC study number 11/NW/0807). All samples used had been collected in the course of routine diagnostic care between 1994 and 2005 and at that time no mutational status analysis was performed on this cohort. All patients included received intensive chemotherapy according to standard UK MRC AML protocols (MRC AML12, AML14 and AML15).

### Tissue microarray construction

Tissue microarrays (TMA) were prepared using an ATA 100 tissue array machine (Chemicon International, Temecula, CA, USA). For this, cylindrical cores 1.5 mm in diameter were taken from paraffin embedded bone marrow trephine samples using a hollow needle and inserted into a precisely spaced ‘recipient’ paraffin wax block. Cores were obtained from areas showing leukemic infiltration on H&E staining. Each TMA contained between 20 and 24 different patient samples. From each patient sample three cores were removed and placed at different sites of the TMA block to maximize separation between related cores within a TMA. Fourteen TMAs were prepared. Sections were cut from the TMA blocks and mounted onto coated glass slides. Each core from each patient contained at least 20% leukemic blasts and was representative of the whole trephine sample.

### P-SYK Y323 measurement

P-SYK Y323 protein expression, assayed by Envision+ disclosed immunopositivity, was measured automatically using an Aperio Scan Scope XT workstation, with ImageScope software (Vista, CA, USA). The Aperio Color Deconvolution v9 algorithm, which measures the optical density per unit area, was used to measure total cellular P-SYK staining, after potential areas of staining artefact such as bone fragments, tissue wrinkles/folding and background debris were excluded from the analysis. P-SYK staining was specific to blasts. Because there is no universal AML blast marker consistently expressed across AML subtypes, dual staining to identify blasts was not possible. Instead, blasts were identified morphologically by pathological examination of the analyzed images. Measurements were performed using the Aperio Image Analysis system. Micrographs were imaged using a Nikon Eclipse 80i microscope, with a Nikon Plan Apo 40_/0.95 air objective and captured using a Nikon DS-F digital camera with NISElements D 3.1 software, with manipulation in CorelDRAWX3. Aperio determines the percentage of positive (brown) staining within the area with haematoxylin-stained tissue and segregates it into 0, 1+, 2+, 3+ based on optical density thresholds for intensity of staining (representative images at each intensity are shown in Supplemental Figure 2A, and 2C respectively, together with total SYK staining in Supplemental Figure 2D. Total, weak (1+), medium (2+) and strong (3+) staining percentage values for each individual case are shown in Supplemental Table 1. Additionally, an H-score (0–300) was calculated from these percentages using the formula H = (3× percentage positivity at 3+) + (2× percentage positivity at 2+) + (1× percentage positivity at 1+), together with a modified H-score in which 1+ staining was aggregated with negative staining, using the formula Mod-H = (3× percentage positivity at 3+) + (2× percentage positivity at 2+) + (0× percentage positivity at 1+) (Supplemental Table 1).

### Statistical analysis

#### Kaplan-meier survival analysis

Kaplan-Meier (K-M) survival analysis was performed using the percentage of area analyzed positive for P-SYK at a range of expression intensity levels (weak/1+ staining, medium/2+ staining, or strong/3+ staining), as measured using the Aperio analysis system. Analysis was performed using cut-offs at either the median, 25^th^, or 75^th^ percentile percentage values, by splitting the patients into two groups with percentage of P-SYK positive area analyzed either equal to or above, or less than the median, 25^th^, or 75^th^ percentile values (Supplemental Table 2). In order to validate the clinical utility of the association of P-SYK staining with outcome, the association was tested using both the H-score and the modified H-score, using the same cutoffs; an H-score is more easily measured in routine clinical practice than the precise percentage of positive cells, whilst the modified H-score emphasizes the distinction between cases with very low to no staining and those with moderate to high staining (Supplemental Table 2).

#### Kruskall-Wallis test and multivariate survival analysis

A Kruskall-Wallis test was performed with medium (2+) and strong (3+) P-SYK positive area grouped less than (1) or equal to/above (2) the 75% percentile together with cytogenetic category and AML FAB subtype. Multivariate survival analysis was performed with Cox regression using either strong (3+) P-SYK positive area or modified H-score together with cytogenetic category, FAB subtype, white cell count (WCC) and age at diagnosis; analysis was performed using a categorical value of strong P-SYK less than (1), or equal to or above (2) the 75% percentile, and a categorical value modified H-score less than (1), or equal to or above (2) the 75% percentile. All statistical analysis was performed using MedCalc version 11.4.40 (Mariakerke, Belgium).

## SUPPLEMENTARY FIGURES AND TABLES



## References

[1] Cortes JE, Kantarjian H, Foran JM, Ghirdaladze D, Zodelava M, Borthakur G, Gammon G, Trone D, Armstrong RC, James J, Levis M (2013). Phase I study of quizartinib administered daily to patients with relapsed or refractory acute myeloid leukemia irrespective of FMS-like tyrosine kinase 3-internal tandem duplication status. Journal of clinical oncology : official journal of the American Society of Clinical Oncology.

[2] Keeton EK, McEachern K, Dillman KS, Palakurthi S, Cao Y, Grondine MR, Kaur S, Wang S, Chen Y, Wu A, Shen M, Gibbons FD, Lamb ML, Zheng X, Stone RM, Deangelo DJ (2014). AZD1208, a potent and selective pan-Pim kinase inhibitor, demonstrates efficacy in preclinical models of acute myeloid leukemia. Blood.

[3] Hahn CK, Berchuck JE, Ross KN, Kakoza RM, Clauser K, Schinzel AC, Ross L, Galinsky I, Davis TN, Silver SJ, Root DE, Stone RM, DeAngelo DJ, Carroll M, Hahn WC, Carr SA (2009). Proteomic and genetic approaches identify Syk as an AML target. Cancer cell.

[4] Miller PG, Al-Shahrour F, Hartwell KA, Chu LP, Jaras M, Puram RV, Puissant A, Callahan KP, Ashton J, McConkey ME, Poveromo LP, Cowley GS, Kharas MG, Labelle M, Shterental S, Fujisaki J (2013). *In Vivo* RNAi screening identifies a leukemia-specific dependence on integrin beta 3 signaling. Cancer cell.

[5] Oellerich T, Oellerich MF, Engelke M, Munch S, Mohr S, Nimz M, Hsiao HH, Corso J, Zhang J, Bohnenberger H, Berg T, Rieger MA, Wienands J, Bug G, Brandts C, Urlaub H (2013). beta2 integrin-derived signals induce cell survival and proliferation of AML blasts by activating a Syk/STAT signaling axis. Blood.

[6] Puissant A, Fenouille N, Alexe G, Pikman Y, Bassil CF, Mehta S, Du J, Kazi JU, Luciano F, Ronnstrand L, Kung AL, Aster JC, Galinsky I, Stone RM, DeAngelo DJ, Hemann MT (2014). SYK is a critical regulator of FLT3 in acute myeloid leukemia. Cancer cell.

[7] Bogusz AM, Baxter RH, Currie T, Sinha P, Sohani AR, Kutok JL, Rodig SJ (2012). Quantitative immunofluorescence reveals the signature of active B-cell receptor signaling in diffuse large B-cell lymphoma. Clinical cancer research: an official journal of the American Association for Cancer Research.

[8] Braselmann S, Taylor V, Zhao H, Wang S, Sylvain C, Baluom M, Qu K, Herlaar E, Lau A, Young C, Wong BR, Lovell S, Sun T, Park G, Argade A, Jurcevic S (2006). R406, an orally available spleen tyrosine kinase inhibitor blocks fc receptor signaling and reduces immune complex-mediated inflammation. The Journal of pharmacology and experimental therapeutics.

[9] Kurosaki T, Takata M, Yamanashi Y, Inazu T, Taniguchi T, Yamamoto T, Yamamura H (1994). Syk activation by the Src-family tyrosine kinase in the B cell receptor signaling. The Journal of experimental medicine.

[10] Zhang J, Billingsley ML, Kincaid RL, Siraganian RP (2000). Phosphorylation of Syk activation loop tyrosines is essential for Syk function. An *in vivo* study using a specific anti-Syk activation loop phosphotyrosine antibody. The Journal of biological chemistry.

[11] Zhang J, Kimura T, Siraganian RP (1998). Mutations in the activation loop tyrosines of protein tyrosine kinase Syk abrogate intracellular signaling but not kinase activity. Journal of immunology.

[12] Byers RJ, Currie T, Tholouli E, Rodig SJ, Kutok JL (2011). MSI2 protein expression predicts unfavorable outcome in acute myeloid leukemia. Blood.

[13] Dunphy CH (2004). Applications of flow cytometry and immunohistochemistry to diagnostic hematopathology. Archives of pathology & laboratory medicine.

[14] Burns JA, Li Y, Cheney CA, Ou Y, Franlin-Pfeifer LL, Kuklin N, Zhang ZQ (2009). Choice of fixative is crucial to successful immunohistochemical detection of phosphoproteins in paraffin-embedded tumor tissues. The journal of histochemistry and cytochemistry: official journal of the Histochemistry Society.

[15] Espina V, Edmiston KH, Heiby M, Pierobon M, Sciro M, Merritt B, Banks S, Deng J, VanMeter AJ, Geho DH, Pastore L, Sennesh J, Petricoin EF, Liotta LA (2008). A portrait of tissue phosphoprotein stability in the clinical tissue procurement process. Molecular & cellular proteomics: MCP.

[16] Fox CH, Johnson FB, Whiting J, Roller PP (1985). Formaldehyde, fixation. The journal of histochemistry and cytochemistry: official journal of the Histochemistry Society.

[17] Holzer TR, Fulford AD, Arkins AM, Grondin JM, Mundy CW, Nasir A, Schade AE (2011). Ischemic time impacts biological integrity of phospho-proteins in PI3K/Akt, Erk/MAPK, and p38 MAPK signaling networks. Anticancer research.

[18] Pham NA, Morrison A, Schwock J, Aviel-Ronen S, Iakovlev V, Tsao MS, Ho J, Hedley DW (2007). Quantitative image analysis of immunohistochemical stains using a CMYK color model. Diagnostic pathology.

[19] Feldman AL, Sun DX, Law ME, Novak AJ, Attygalle AD, Thorland EC, Fink SR, Vrana JA, Caron BL, Morice WG, Remstein ED, Grogg KL, Kurtin PJ, Macon WR, Dogan A (2008). Overexpression of Syk tyrosine kinase in peripheral T-cell lymphomas. Leukemia.

[20] Kuno Y, Abe A, Emi N, Iida M, Yokozawa T, Towatari M, Tanimoto M, Saito H (2001). Constitutive kinase activation of the TEL-Syk fusion gene in myelodysplastic syndrome with t(9:12)(q22;p12). Blood.

[21] Tomasson MH, Xiang Z, Walgren R, Zhao Y, Kasai Y, Miner T, Ries RE, Lubman O, Fremont DH, McLellan MD, Payton JE, Westervelt P, DiPersio JF, Link DC, Walter MJ, Graubert TA (2008). Somatic mutations and germline sequence variants in the expressed tyrosine kinase genes of patients with de novo acute myeloid leukemia. Blood.

[22] Irish JM, Hovland R, Krutzik PO, Perez OD, Bruserud O, Gjertsen BT, Nolan GP (2004). Single cell profiling of potentiated phospho-protein networks in cancer cells. Cell.

[23] Skavland J, Jorgensen KM, Hadziavdic K, Hovland R, Jonassen I, Bruserud O, Gjertsen BT (2011). Specific cellular signal-transduction responses to *in vivo* combination therapy with ATRA, valproic acid and theophylline in acute myeloid leukemia. Blood cancer journal.

