# Supplementary material for: Increased SYK activity is associated with unfavorable outcome among patients with acute myeloid leukemia
Source: Oncotarget. 2015 Aug 11;6(28):25575–87. doi: 10.18632/oncotarget.4669 (PMC4694851; doi:10.18632/oncotarget.4669)
Supplement: Supplementary file 1 [file oncotarget-06-25575-s001.pdf]

SUPPLEMENTARY FIGURES AND TABLES

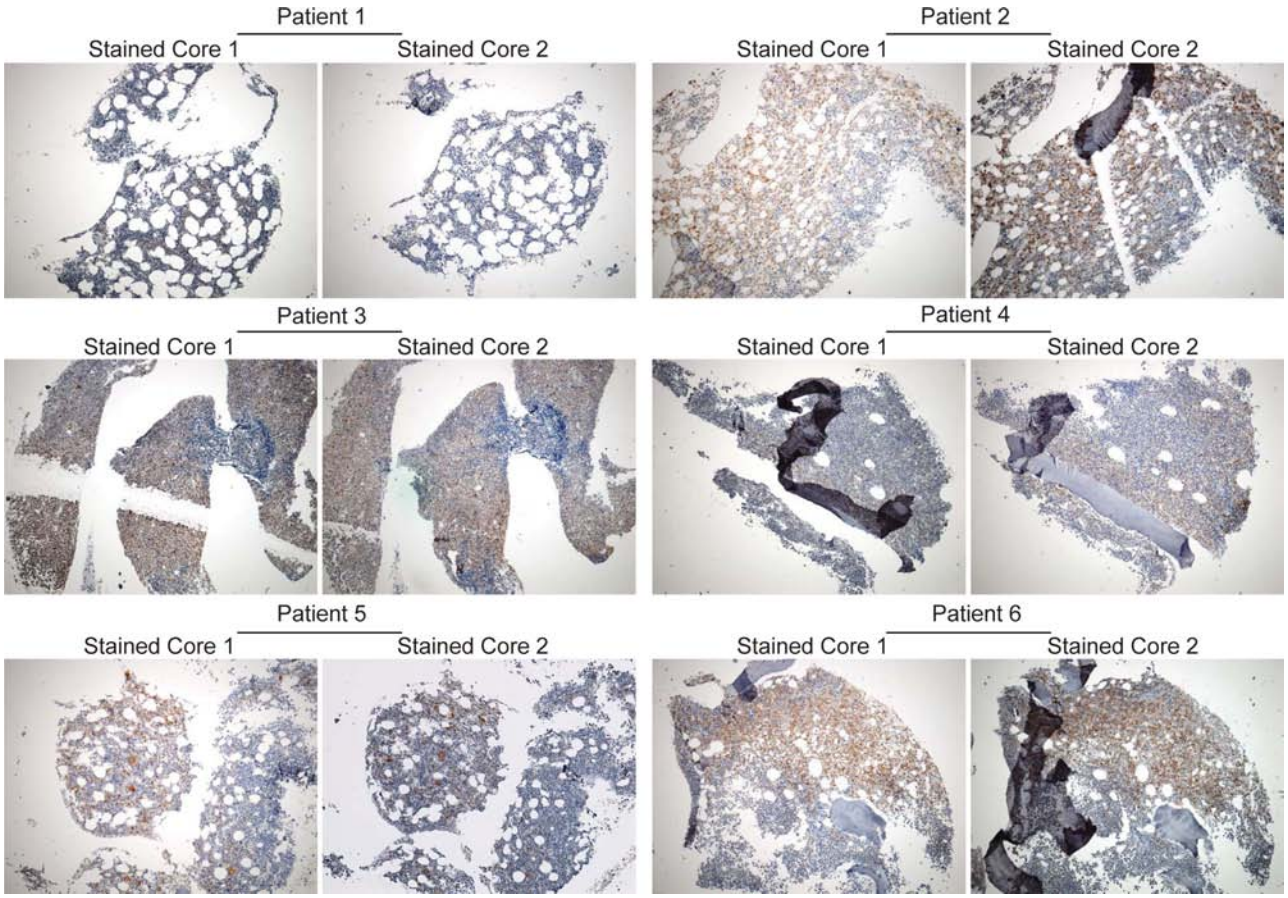

Supplementary Figure S1: Reproducibility of P-SYK staining accross duplicate staining runs on 6 patient samples. Slides were counterstained with Harris haematoxylin. Micrographs were imaged using a Nikon Eclipse 80i microscope.

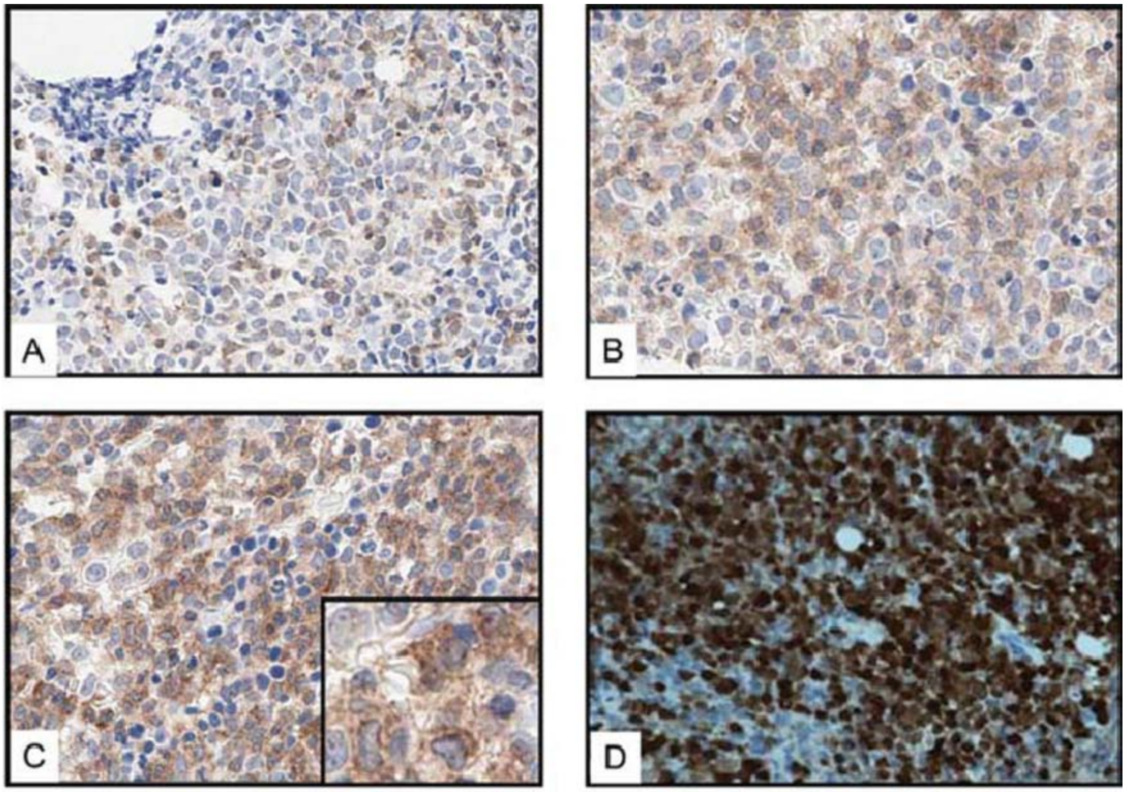

Supplementary Figure S2: P-SYK staining intensities. Representative images for **A.** weak, **B.** medium, **C.** strong P-SYK (Y323) intensity staining, and **D.** total SYK intensity staining used in Aperio analysis are shown. Original magnification x400; sections were stained with diaminobenzidine and H&E. Micrographs were imaged using a Nikon Eclipse 80i microscope, with a Nikon Plan Apo 40_/0.95 air objective and captured using a Nikon DS-F digital camera with NISElements D 3.1 software, with manipulation in CorelDRAWX3.

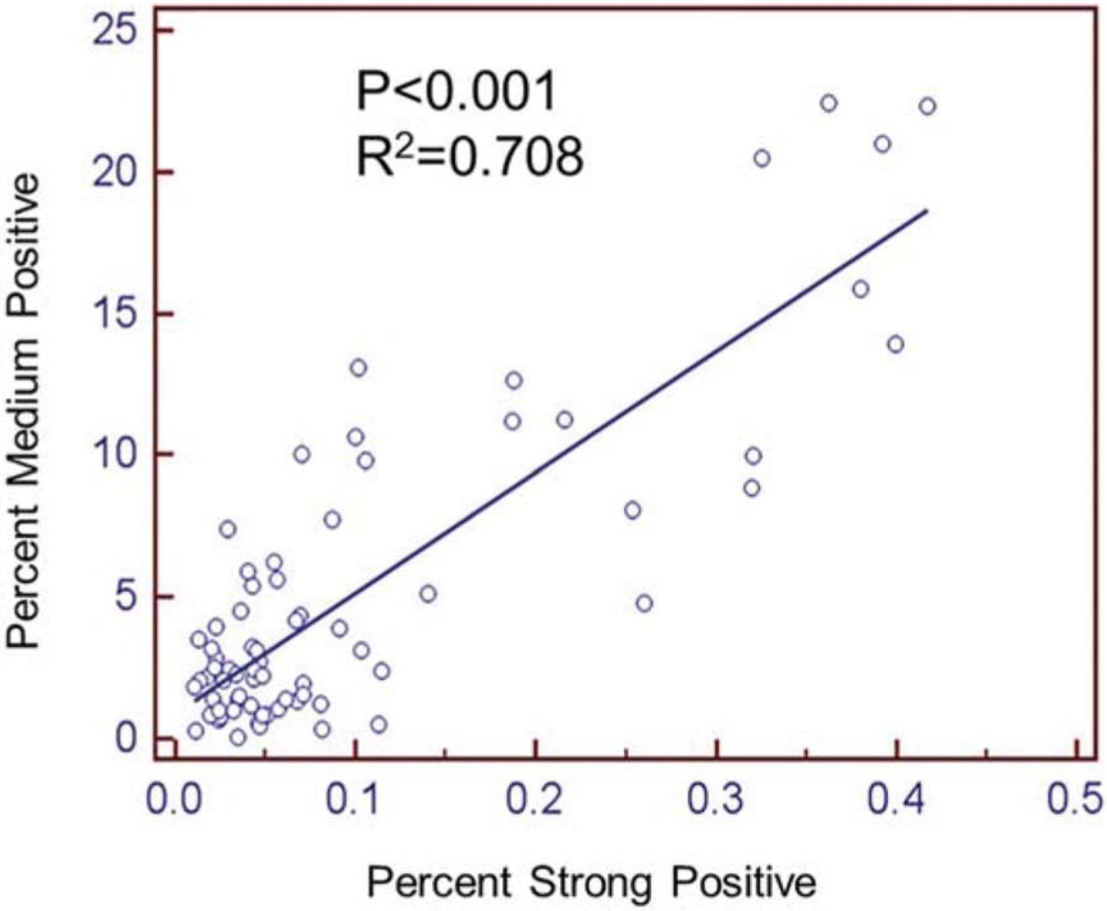

Supplementary Figure S3: Correlation of paired P-SYK staining intensities. Scatterplot of the percentage values of medium and strong p-SYK staining intensities, indicating strong correlation between the two. P and R^2 values are shown.

Supplementary Table S1. Clinical Information and p-SYK Staining Level for AML Patients.

Supplementary Table S2. Cut-off values of percentile groups for staining intensity and H-scores

Percentile group	Weak Staining (1+)	Medium Staining (2+)	Strong Staining (3+)	Total Staining	H-Score	Modified H-Score
Median	59.07	2.80	0.05	63.71	67.80	5.67
25th percentile	50.84	1.39	0.03	52.95	54.76	2.88
75th percentile	65.48	7.11	0.10	73.34	79.64	14.33

The percentage values used to delineate percentile groups of cells with the indicated staining intensity, total staining and H scores are shown.

Supplementary Table S3. Distribution and correlation of staining intensities

	Weak (1+)	Medium (2+)	Strong (3+)
Distribution, %	13.96–80.06	0.07–22.45	0.01–0.4
R^2 weak	–	0.22*	0.10†
R^2 medium	0.22*	–	0.71*
R^2 strong	0.10†	0.71*	–

R^2 indicates the coefficient of determination for the staining intensity groups indicated in the row and column headers.
*$P < 0.001$
†$P = 0.007$
